# Synthesis and Characterization of the Mixed Metal Oxide of ZnO-TiO_2_ Decorated by Polyaniline as a Protective Film for Acidic Steel Corrosion: Experimental, and Computational Inspections

**DOI:** 10.3390/ma15217589

**Published:** 2022-10-28

**Authors:** May Ahmed Al-Masoud, Mai M. Khalaf, Mohamed Gouda, Van-Duong Dao, Ibrahim M. A. Mohamed, Kamal Shalabi, Hany M. Abd El-Lateef

**Affiliations:** 1 Department of Chemistry, College of Science, King Faisal University, Al-Ahsa 31982, Saudi Arabia; 2 Department of Chemistry, Faculty of Science, Sohag University, Sohag 82524, Egypt; 3 Faculty of Biotechnology, Chemistry and Environmental Engineering, Phenikaa University, Hanoi 10000, Vietnam; 4 Department of Chemistry, College of Science and Humanities in Al-Kharj, Prince Sattam bin Abdul-Aziz University, Al-Kharj 11942, Saudi Arabia; 5 Chemistry Department, Faculty of Science, Mansoura University, Mansoura 35516, Egypt

**Keywords:** polymeric nanocomposite, corrosion protection, electrochemical studies, theoretical and computational research, petroleum industry

## Abstract

In this work, the preparation, characterization, and evaluation of a novel nanocomposite using polyaniline (PANi) functionalized bi-metal oxide ZnO-TiO_2_ (ZnTiO@PANi) as shielding film for carbon steel (CS)-alloy in acidic chloride solution at 298 K was studied. Different spectroscopic characterization techniques, such as UV-visible spectroscopy, dynamic light scattering (DLS), X-ray photoelectron spectroscopy (XPS), and Fourier transform infrared spectroscopy (FTIR) approaches, as well as other physicochemical methods, such as X-ray diffraction (XRD), high-resolution transmission electron microscopy (HR-TEM), and field emission scanning electron microscope (FESEM), were used to describe the produced nanocomposites. The significance of these films lies in the ZnO-TiO_2_ nanoparticle’s functionalization by polyaniline, a material with high conductivity and electrochemical stability in acidic solutions. The mechanistic findings of the corrosion inhibition method were obtained by the use of electrochemical methods including open-circuit potentials (OCP) vs. time, potentiodynamic polarization (PDP), and electrochemical impedance spectroscopy (EIS). The results indicate that the synthesized ZnTiO@PANi is a powerful acidic corrosion inhibitor, and its inhibition effectiveness is 98.86% in the presence of 100 ppm. Additionally, the charge transfer resistance (*R*_p_) value augmented from 51.8 to 432.7, and 963.7 Ω cm^2^ when the dose of PANi, and ZnTiO@PANi reached 100 ppm, respectively. The improvement in *R*_p_ and inhibition capacity values with an increase in nanocomposite dose is produced by the nanocomposite additives covering a larger portion of the surface, resulting in a decrease in alloy corrosion. By identifying the probable regions for molecule adsorption on the steel substrate, theoretical and computational studies provided significant details regarding the corrosion mitigation mechanism. The possibility of substituting old poisonous small substances with inexpensive and non-hazardous polymeric materials as shielding layers for utilization in the oilfield sectors is an important suggestion made by this research.

## 1. Introduction

Carbon steel is a requisite metal alloy for several applications in a wide range of industries, such as railways, buildings, oil, gas industry, and other fields. However, carbon steel is also sensitive to deterioration in extreme conditions, which demands the replacement of many parts and leads to massive losses in time and money in order to repair the resulting damages. So, this issue attracts the attention of researchers and scientists to find practical solutions for this vital problem [1,2]. Many techniques were employed to avoid or reduce the influence of corrosion, using corrosion inhibitors is one of the best functional economical methods used to enhance the mechanical properties and resistance of metals against corrosion [3]. Applying inhibitors in corrosion protection owns high technological importance due to the reasonable cost and the straight implementation methodology [4]. Previously, utilized inhibitors were either toxic or active at high concentrations so, it was necessary to alternate these toxic inhibitors with more safe and friendly ones [5,6,7].

Recently, conductive polymers (CPs) that comprise π-conjugation in their backbones, such as PANi, PTh, and PPy, achieved considerable importance in metallic corrosion prevention even in damaged areas where metal is in contact with aggressive environs [8]. In recent years, polyaniline (PANi), a significant conducting polymer, saw widespread use. The conjugated-electron system described in PANi practically increases conductivity, opening the door to its many applications as a battery electrode material, biosensors, corrosion prevention techniques, as well as the large molecular size that will improve inhibition through metal surface coverage [9,10,11].

On the other hand, it is found that the properties of the pristine conductive polymer are insufficient in many cases because they cannot prevent the penetration of aggressive ions through the polymer pores [12]. As a result, new approaches were being researched to address the drawbacks of pure polymeric materials. One of these approaches involves using a variety of inorganic fillers, such as metal oxides, within the polymer matrix to close pores and prevent aggressive ions from penetrating the material.

For instance, nanoparticles (NPs), such as alumina, titania, and silica [13] are common fillers that are used in corrosion prevention. The development of polymer nanocomposites based on CPs and metal oxides in corrosion protection improves the mechanical and physicochemical features of the polymeric matrix, such as barrier effect, adhesion, and lower porosity because the organic and inorganic properties of the materials are incorporated more efficiently at the micrometer and nanometer scales [14]. Jiang et al. [15] improved the corrosion resistance and wettability of (TiO_2_) by introducing a film of polydopamine epoxy resin between the TiO_2_ layer and metal surface. They deduced that the merged coating with polydopamine improves the adhesion of titania film to the studied metal. Shen et al. [16] mentioned that polymer\TiO_2_ nanocomposite coatings show excellent performance against corrosion owing to the protective barrier of the polymer and the further cathodic protection that is generated under UV light, as TiO_2_ has a good photic response [17]. It was suggested that the use of TiO_2_ in coatings, provides a form of cathodic protection in order to minimize the tendency to corrosion [16,18]. Additionally, ZnO was introduced as a promising material in different applications [19,20,21]. Therefore, this work introduces mixed oxide in addition to polymer content inside one composite material.

There are small previous publications on the use of bi-metal oxide/polymer nanocomposites as protective films for steel corrosion such as polyaniline functionalized ZnO-SiO_2_ [22]. In order to create ZnO-TiO_2_/PANi nanocomposite and evaluate its corrosion protection capabilities, double-metal zinc-titanium (ZnO-TiO_2_) oxide was used as a fundamental ingredient in this work. The prepared nanocomposite systems were categorized using several spectroscopic approaches, including XRD, UV-vis, FTIR, HR-TEM, FESEM, DLS, and XPS techniques. Utilizing the E_OCP_, EIS, and PDP methods, the corrosion protection characteristics of the synthesized ZnO-TiO_2_@PANi nanocomposite were examined. The adsorption energy and arrangement, as well as quantum chemical aspects, are discussed using MC simulations and DFT calculations so as to additionally clarify the mechanism of the inhibitory process.

## 2. Materials and Methods

### 2.1. Materials, Solutions, and Sample Preparation

In this study, 99% pure Zn(NO_3_)_2_·6H_2_O (zinc nitrate hexahydrate), absolute ethyl alcohol (99%), sodium dodecyl sulfate (SDS) (99.0%), potassium hydroxide (pellets, ACS reagent 85%), ammonia (NH_3_) (33%), urea (99%, pellets), 37% hydrochloric acid (HCl, Merck), aniline (99.5%), potassium persulfate (KPS) (K_2_S_2_O_8_) (98%), and titanium (IV) isopropoxide (Ti(OCH(CH_3_)_2_)_4_) were used. All Sigma-A.R Aldrich’s grade compounds were used without further purification.

### 2.2. Preparation of ZnO·TiO_2_ Nanoparticles

#### 2.2.1. Fabrication of ZnO-Nanoparticles

By the sol-gel method, ZnO nanoparticles were prepared; a stoichiometric quantity of zinc nitrate hexahydrate was dissolved in 20.0 mL of double-distilled H_2_O. The mixed solution was stirred at 450 rpm with a magnetic stirrer for a few minutes and kept under continuous stirring until complete dissolution. A hydrolysis procedure was carried out by dissolving 0.3 M of potassium hydroxide. Under constant stirring, 1.50 M of the (SDS + urea) solution was added to the same Zn beaker while maintaining the mass ratio of the fuel: Zn near the unity. The fuel plays a major role in slowing down the nanoparticle’s growth rate. Using a specified quantity of (0.3 M) potassium hydroxide solution that was added gradually while stirring, the pH of the resultant solution was adjusted at pH = 7–8 to produce Zn Sol. After then, the mixture was vigorously stirred for another two hours [23]. The resulting solution was then subjected to 40 °C sonication for 40 min. Later, the solution was filtrated and rinsed with bi-distilled water. The acquired precipitate was desiccated in a vacuum oven at 110 °C for three hours, then grind well using a mortar to get ZnO nanoparticles (Figure S1; supporting information).

#### 2.2.2. Preparation of ZnO-TiO_2_ Binary Metal Oxide Nanoparticles

A precise amount of iso-propanol and titanium (IV) isopropoxide were added to a separate beaker. Following the addition of the fuel solution (SDS + urea) to the initial mixture, the mixture was continuously stirred until the dissolution was complete. By gently adding 33% NH3 while stirring, the pH of the obtained TiO_2_ solution was determined to be 7.5–8. The mixture was then vigorously stirred for an additional two hours to produce TiO_2_ Sol. Finally, the TiO_2_ Sol. was dropped into the ZnO Sol. while it was being vigorously stirred for a further hour. The resulting ZnO-TiO_2_ solution underwent 40 °C ultra-sonication for 40 min before being filtered and bi-distilled water washed over it. The generated binary mixed oxides were thoroughly ground to form ZnO-TiO_2_ nano-powders after being dried at 130 °C for three hours (Figure S2; supporting information).

#### 2.2.3. Preparation of ZnO·TiO_2_/PANI Nanocomposites

Operating the method of oxidation in situ polymerization, a ZnO-TiO_2_/PANI nanocomposite was made by dispersing 0.7 g of the previously synthesized ZnO.TiO_2_ powder in (23:2) *v*:*v* of bi-distilled H_2_O and EtOH, as per the methods described in [11]. The mixture was swirled for a short time at (450 rpm), and then it was ultra-sonicated for 30 min at 35 °C (labeled as Sol. A). A 2.20 mL aniline solution in 1.0 M of HCl was dispersed for 25 min in another beaker. Then, while continuously stirring, 2.30 g of KPS was added to the initial mix (Sol. B). After obtaining, a precipitate of emeraldine salt that was blackish-green in color (Sol. B) was slowly added to (Sol. A) while stirring. This process was continued for three hours. This combined solution was placed in an ice bath for 12 h at a low temperature (5 °C). It was then filtered, cleaned with (1.0 M) HCl, and dried in a vacuum oven for 3.0–4.0 h at 65 °C. To achieve an emeraldine base of polyaniline, the obtained precipitate was pickled with (1.0 M) ammonia while stirring and the pH was attuned to 8. The mix was agitated continuously for 12 h before being filtered and repeatedly rinsed with distilled water. Once the black precipitate formed, it was dehydrated in a vacuum oven at 338 K for 4.0 h before being ground into powder. ZnTiO/PANi was used to code this sample (Figure S3; supporting data).

### 2.3. Characterization Techniques

Scanning electron microscopy (SEM JEOL, JSM-6360 LA, Tokyo, Japan) was applied to know the morphological image of the prepared ZnTiO@PANi composite. Additionally, a transmission electron microscope (TEM) in a Jeol-1230 electron microscope was applied to affirm the particle size of the prepared ZnTiO@PANi composite. The crystallinity of the synthesized ZnTiO@PANi composite was studied through X-ray diffraction (XRD; TD-3500 diffractometer, Dandong Mastery Technology Co., Ltd., Dandong, China) at room temperature with Ni-filtered CuKα radiation (λ = 1.5418 Å), at 40 kV and 30 mA. N_2_ adsorption-desorption isotherm was additionally used to know the surface area and porosity of the prepared ZnTiO@PANi composite using Brunauer–Emmett–Teller (BET) at 77 K (Tristar II 3020 version 3.02, Norcross, GA, USA). The UV–vis spectroscopy was analyzed by a Shimadzu UVPC-1800 spectrophotometer (Kyoto, Japan), using a 5.0 mm path length quartz and was recorded between 200 and 750 nm wavelengths. The FTIR spectroscopy using BRUKER equipment was studied to describe the chemical bonds of the prepared composite. The surface chemistry of the prepared ZnTiO@PANi composite sample was tested via XPS (XPS, Escalab 250Xi, Thermo Scientific, Waltham, MA, USA). Thermal analysis equipment from New Castle, DE 19720, USA was utilized to investigate the thermal stability of the ZnTiO@PANi composite by heating it to 1000 °C under the flow of O_2_. Additionally, the dynamic light scattering technique (DLS)-nano Zetasizer (model: Zetasizer nano ZS90 Malvern Instruments, Cambridgeshire, UK) was utilized to determine the particle size distribution of the prepared ZnTiO@PANi composite.

### 2.4. Electrochemical Experiments

The advantage of the electrochemical method is that it permits speedy evaluation of the metal rate corrosion without waiting for a long-time for gravimetric examinations. These techniques need an electrochemical cell that restricts a working electrode (CS-alloy), a reference silver/silver chloride electrode (Ag/AgCl_(sat)_), and an auxiliary electrode (Pt-wire). The electrodes are connected to a Gamry Galvanostat/Potentiostat/ZRA electrochemical workstation, which in turn is connected to a computer-controlled by ECehm Analyst software from Gamry. When the potential for an open circuit (*E*_OCP_) was reached in the analyzed corrosive solution, electrochemical experiments were conducted there. The PDP curves are examined at a temperature of 298 K, a scan rate of 0.2 mV/s, and a potential range of −250 mV and +250 mV vs. *E*_OCP_. The AC signal was used to conduct steady-state electrochemical impedance spectroscopy (EIS) throughout a frequency range of 100 kHz to 0.1 Hz with an amplitude of 10 mV.

### 2.5. Preparation of the Specimens for the Corrosion Inhibition Experimentations

The low carbon steel alloy (CS-alloy) employed as the working electrode (WE) had the following chemical composition (ASTM) (wt%): 0.29% C, 0.15% Cr, 0.88% Mn, 0.034% P, 0.039% S, 0.18% Si, and the remainder was Fe as shown in Table 1. The CS-alloy with a dipped area of 0.50 cm^2^ in epoxy resin served as the WE for the measurements of corrosion. The samples were involuntarily scraped with a sequence of silicon carbide sheets of varying degrees, starting with a rough one (600), and progressing gradually to the premium (1800) grade. Following a thorough cleaning with bi-distilled H_2_O, the sample was only degreased with acetone before being placed inside the cell. An acidic chloride solution (1.0 M HCl + 3.5% NaCl) was utilized as a corrosive environment and was prepared by diluting the 37.0% hydrochloric acid (Merck) with a 3.5% sodium chloride solution. A standard solution of 50,000 mg/L was made in 50.0 mL of the aggressive solution using 1.0 g of the solid ZnTiO@PANI. For the corrosion inhibition experiments, the applied dosages of 10.0, 20.0, 40.0, 60.0, and 100.0 ppm were created by adequate dilution.

### 2.6. Computational Information

Utilizing DNP 4.4 and the B3LYP-functional basis set carried out in the Dmol3 module of the BIOVIA Materials Studio 2017, Dassault Systèmes, France for DFT calculations, the energy minimization of the examined PANi, ZO@PANi, and ZnTiO@PANi compounds in aqueous conditions was evaluated [24]. The outcomes attained from DFT calculations, such as the highest occupied molecular orbital (*E*_HOMO_), the lowest unoccupied molecular orbital (*E*_LUMO_), the gap energy (Δ*E*), hardness (*η*), electronegativity (*χ*), global softness (*σ*), electrophilicity index (*ω*), and ∆*E*_back-donation_, dipole moment (*µ*), and the number of electrons transferred (Δ*N*), were examined and calculated as follows [25]:

(1) $$\chi =\frac{-E_{HOMO}-E_{LUMO}}{2}$$

(2) $$\eta =\frac{1}{\sigma }=\frac{E_{LUMO}-E_{HOMO}}{2}$$

(3) $$\omega =\frac{\chi^{2}}{2\eta }$$

(4) $$\Delta N=\frac{\varphi -\chi_{inh}}{2(\eta_{Fe}-\eta_{inh})}$$

(5) $$\Delta E_{back-donation}=\frac{-\eta }{4}$$

where *χ_inh_* signifies the inhibitor electronegativity, *φ* is the Fe (110) function work, *η_Fe_* and *η_inh_* are the chemical hardness for Fe (0 eV) and additives, respectively.

Operating the adsorption locator module in the BIOVIA Materials Studio 2017, Dassault Systèmes, France revealed the correct adsorption configurations of the PANi, ZO@PANi, and ZnTiO@PANi compounds on the iron (110) interface for MC simulations [26]. First, the COMPASS force field was operated to optimize the adsorbate molecules [27]. Subsequently, in a simulation box (37.24Å × 59.81Å × 37.24Å), the studied inhibitors, chloride ions, hydronium ions, and water molecules were successfully adsorbed onto the surface of Fe(110) [28]. 

## 3. Results and Discussion

### 3.1. Characterization of the Prepared Material

#### 3.1.1. SEM, TEM, XRD, and DLS Investigations

**Figure 1:**
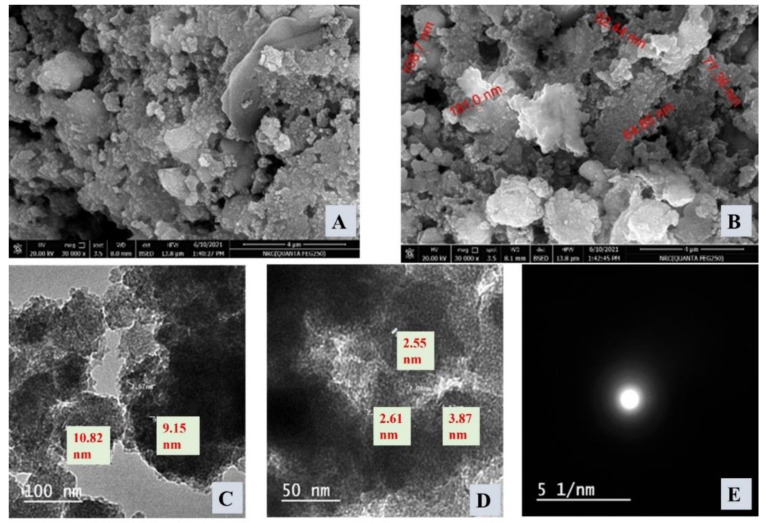
(**A**) SEM image of ZnTiO@PANi nanocomposite, (**B**) SEM image of ZnTiO@PANi nanocomposite in another investigation area, (**C**) TEM of ZnTiO@PANi nanocomposite, (**D**) HR-TEM of ZnTiO@PANi nanocomposite at higher magnification, and (**E**) SAED of ZnTiO@PANi nanocomposite.

The SEM images of the presented ZnTiO@PANi composite were shown in Figure 1. The morphology is quite irregular and appeared as globular agglomerate particles of ZnTiO@PANi of a changeable size alternating from 52.44 to 181 nm. There is no clear porosity that could be detected which indicates the filling of pores by the formation of composite: organic/oxide composite. Additionally, TEM, HR-TEM, and SAED images were described in Figure 1C–E, respectively. Some crystalline structures could be seen, which could be attributed to the oxide part of the prepared composite: ZnO/TiO_2_. In addition, the ring circles sequence in the displayed SAED image was not clear in ZnTiO@PANi, owing to the incorporation of the oxide part into the polymer part, i.e., the PANi matrix, which could reduce the crystallinity degree. Thus, the SEM, besides TEM images, confirmed that the prepared ZnTiO@PANi composite material has a heterogeneous morphology containing crystalline (oxide) and amorphous (polymer) parts. 

**Figure 2:**
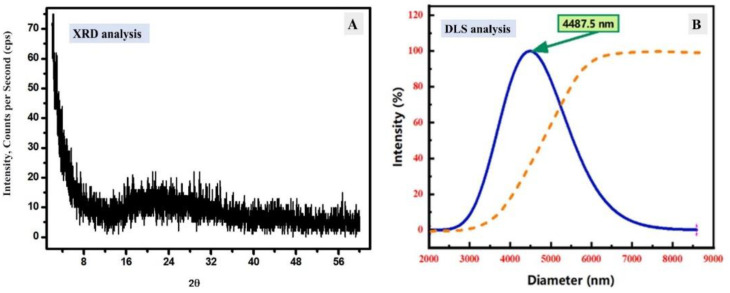
(**A**) XRD analysis of ZnTiO@PANi composite, (**B**) DLS analysis of ZnTiO@PANi nanocomposite.

Figure 2A displays the XRD pattern of the prepared ZnTiO@PANi and shows the amorphous character, which dominated the XRD character. This amorphous character could be attributed to the existence of polymer content as a major content. The main broad peak was around 2θ = 20°, which is related to the polymer cross chains. Moreover, there are small peaks of ZnO and TiO_2_ at 31.65° and 25.49°. To conclude, the XRD analysis displayed the amorphous structure of the prepared ZnTiO@PANi composite without clear crystalline peaks. The DLS distribution curve of the synthesized ZnTiO@PANi composite was displayed in Figure 2B. The shown distribution exhibits a broad and symmetrical hydrodynamic size distribution enlarged at 4487.5 nm. This ideal symmetry distribution explains the uniformity of fabricated composite and their dispersion in the investigated medium, due to the stable electric dipole layer that adhered around it [29]. 

#### 3.1.2. FT-IR and UV–Visible Spectroscopy 

**Figure 3:**
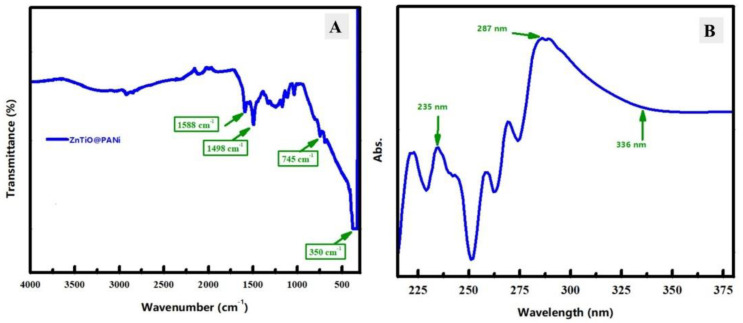
(**A**) FT-IR spectroscopy of the investigated ZnTiO@PANi composite, (**B**) UV–visible spectroscopy in the aggressive solution of the examined ZnTiO@PANi composite.

FTIR analysis of the synthesized ZnTiO@PANi composite was investigated to study the chemical bonds and functional chemical groups of the prepared ZnTiO@PANi composite (Figure 3A). The FTIR spectrum curve shows remarkable bands of the polymer contents at 1588 cm^−1^ and 1498 cm^−1^ that could be assigned to the stretching vibration of C=C and C=N, respectively [30]. Moreover, the small FT-IR peak at 745 cm^−1^ could be related to the anti-symmetric stretching for Ti–O–Ti bonds. The shift of the FT-IR peak of the Ti–O–Ti bond could be due to the interaction between polymer function bonds and transition metal oxide bonds. Additionally, a prominent peak could be noticed at 350 cm^−1^, which is due to the out-of-plane C-H bending mode of the aromatic ring of PANi [31]. Therefore, the FT-IR bands indicate the effective incorporation of mixed metal oxides into the polymer matrix.

Figure 3B exposes the UV–vis spectrum curve of the studied ZnTiO@PANi composite, in order to examine the optical properties in the corrosive medium. The ZnTiO@PANi composite spectrum has a notable absorption band at 235 nm, which could be assigned to the π-π* transition of the benzenoid ring in the polymeric content. Additionally, another peak is observed around 287 nm, which could be ascribed to the interaction between oxide and PANi [8] and the absorption values begin to decline from 336 nm, approximately. Therefore, spectroscopic analyzes, including FT-IR and UV-vis could indicate the successful interaction between the oxide part and polymer content to finally design the ZnTiO@PANi composite. 

#### 3.1.3. Surface Chemistry Analysis 

**Figure 4:**
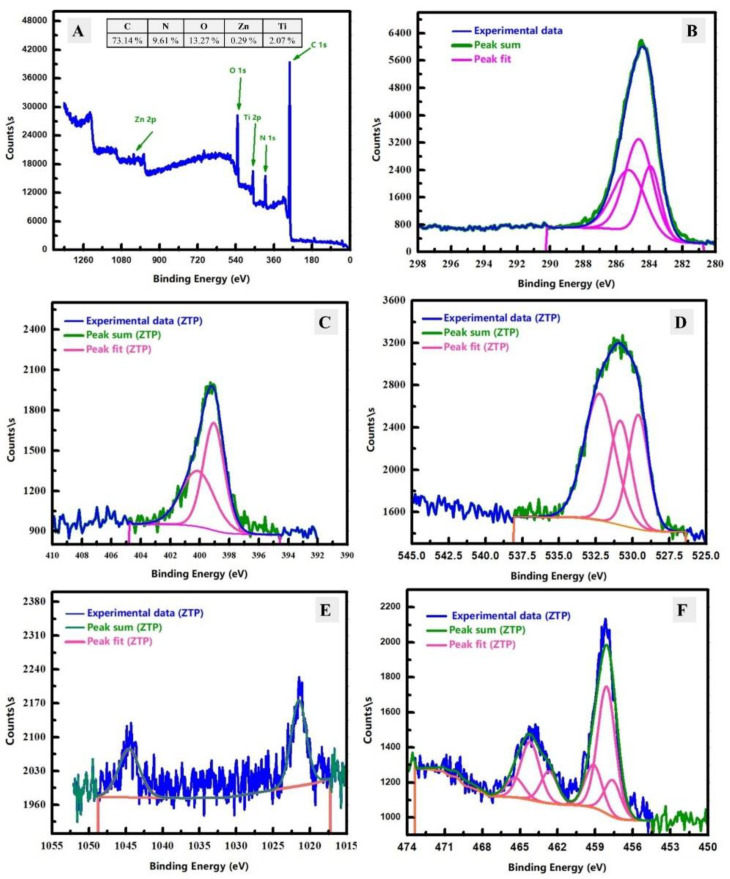
XPS analysis of the prepared ZnTiO@PANi composite; (**A**), and the core-level spectra of C 1 s; (**B**), N 1s (**C**), O 1s; (**D**), Zn 2p (**E**), and Ti 2p; (**F**).

XPS analysis was applied to understand the surface chemistry of the ZnTiO@PANi composite as exhibited in Figure 4. The XPS examination at a fast scan of the fabricated ZnTiO@PANi composite was displayed in Figure 4A as well as the binding energy areas of its constituents (N, O, C, Zn, and Ti) by a good-rate spectrum were demonstrated in Figure 4B–F, respectively. The XPS survey peaked at 285 eV, 401.08 eV, 531 eV, 1022.08 eV, and 459 eV which indicate the existence of C 1s, N 1s, O 1s, Zn 2p, and Ti 2P [32]. This result could confirm the presence of both polymer and oxide at the surface of the prepared composite. The C, N, O, Zn, and Ti regions could be further scanned by the slow scan as displayed in Figure 4B–F, respectively. In the case of C 1s (Figure 4B), the deconvoluted peaks of the C1s spectrum have three peaks at 284 eV, 284.68 eV, and 284.89 eV, which could be assigned to different carbon bonds in the composite: C=C, C-N, and C=O, respectively [22,33]. For N-fitting analysis (Figure 4C), the presence of deconvoluted two peaks at 399.21 eV and 399.62 eV could be attributed to N-content in –C=N- and the O–Ti–N [34]. Figure 4D shows the deconvoluted peaks of the O1s spectrum, which have three peaks located at 529.61 eV, 530.84 eV, and 532.25 eV, which signify the three different types of O-bonds; O-H, Ti-O, and C=O [34]. After that, the Zn 2p area was studied and shown in Figure 4E, which has two clear peaks at 1021.53 eV and 1044.55 eV, which could be due to Zn 2p3/2 and Zn 2p1/2, respectively. Additionally, the separation between Zn 2p3/2 and 2p1/2 peaks was found around 23.02 eV, which indicated the Zn is Zn(II) in the prepared composite [33]. At last, Figure 4F shows the Ti 2p binding energy region, which contains peaks at 464.45 eV and 458.02 eV. These peaks are related to Ti 2P1/2 and Ti 2P3/2, and the splitting between them is 6.43 eV, which designates the Ti has IV valence in the fabricated composite [34]. The atomic % percentages of C, N, O, Zn, and Ti were found at 73.14%, 9.61%, 13.27%, 0.29%, and 2.07%, respectively, as displayed in the inset table in Figure 4A. Therefore, carbon is the main content of the prepared composite, which indicates that polymeric content is the major content at the surface of the prepared composite. Consequently, the XPS analysis endorses the surface chemistry of the synthesized composite that has C as a major content in addition to O, N, Zn, and Ti.

#### 3.1.4. BET Surface Area and Thermal Analysis

**Figure 5:**
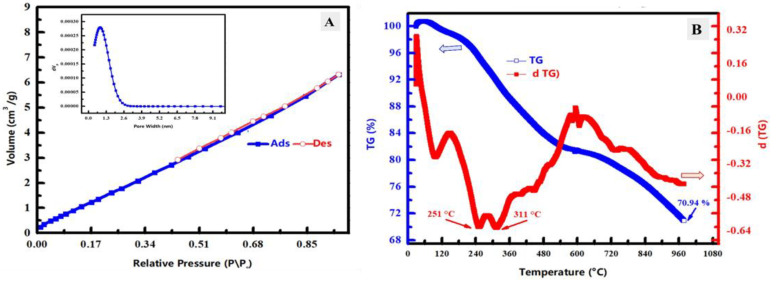
(**A**) Nitrogen adsorption–desorption analysis of the prepared ZnTiO@PANi composite including the pore width analysis in the inset of the figure. (**B**) TGA and DTG analysis of the prepared ZnTiO@PANi composite.

N_2_ adsorption–desorption isotherm of the prepared ZnTiO@PANi composite was shown in Figure 5A to describe the porosity of the composite in addition to the calculation of BET surface area at 77 K. According to the found isotherm, it could be classified as type IV with H3 hysteresis loop based on traditional IUPAC classification. The BET surface area value of the prepared ZnTiO@PANi composite was estimated and found at 8.75 m^2^/g along with a total pore volume of 9.77 × 10^−3^ cm^3^/g, besides a mean pore width of 4.46 nm. The prepared ZnTiO@PANi composite has a low surface area because of filling the PANi matrix pores with bi-oxide content. Therefore, the introduction of metallic parts decreased the estimated BET surface area. Although the BET value of the prepared ZnTiO@PANi composite was low, the pores might be filled with crystalline oxides, which could be a strict inhibitor against material deterioration or oxidation. 

TGA and DTG investigations were studied for the prepared ZnTiO@PANi composite as revealed in Figure 5B. The weight loss was found around 70.94% at the last investigated temperature (977 °C) owing to the evaporation or decomposition of the combined PANI polymer molecules, which confirms the successful synthesis of the composite having polymer and metallic contents. There are three main stages of DTG that could be noticed. The first one is around 105 °C, which could be related to adsorbed humidity. The second and third peaks appeared at 251 °C and 311 °C, respectively, which could be owing to the decomposition of chemical bonds among the combined PANi and the crystallized solvent. Furthermore, the organic contents decomposition of PANI could be seen as several peaks with temperature raising. These diverse PANI evaporation peaks could be caused by the structure of PANI, which contains a variety of O-functional groups and alkyl chains [35]. As a result, TGA and DTG analysis approves the effective synthesis of ZnTiO@PANi composite incorporating the PANI polymer and metal oxide part.

### 3.2. Corrosion protection studies 

#### 3.2.1. Open Circuit Potential vs. Time and PDP Studies

**Figure 6:**
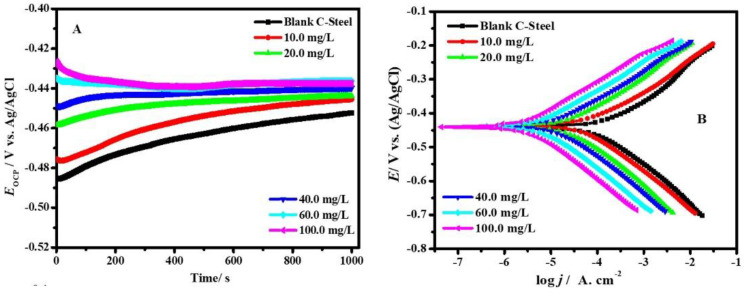
*E*_OCP_ vs. *t* (**A**) and PDP graphs (**B**) of CS-alloy corrosion in the blank molar HCl containing 3.5% NaCl and with adding various doses of ZnTiO@PANi at 25 °C.

*E*_OCP_ vs. *t* (A) and PDP diagrams (B) of the corrosion of CS-alloy in the blank molar HCl containing 3.5% NaCl and with the addition of diverse concentrations of ZnTiO@PANi at 298 K is presented in Figure 6A,B. It is observable from Figure 6A that the *OCP* of the CS-alloy in the investigated corrosive media tendency initially is moderately in the positive trend, displaying an increase to a minor stage. In the presence of the ZnTiO@PANi composite, the *E*_OCP_ values shifted to more positive potential values during the immersion compared with the uninhibited system. This could be designated by the adsorption of ZnTiO@PANi composite on the interface of CS-alloy/solution. Then, the values of *E*_OCP_ tended to be stable, signifying that the adsorption and desorption ZnTiO@PANi composite had gotten a dynamic steadiness [28]. This recommends that the steel anodic reaction in the studied aggressive solution was affected more strongly in the system comprising the ZnTiO@PANi composite. The *E*_OCP_ outcomes suggested that the ZnO/TiO_2_ nanoparticle functionalized by PANi has strong adsorption on the steel surface.

Figure 6B shows the potentiodynamic curves of CS-alloy corrosion in the uninhibited and inhibited with the addition of different concentrations of ZnTiO@PAN at 25 °C, commonly, the cathodic and anodic branches of the Tafel plots. It is clear that the presence of the ZnTiO@PAN composite causes a marked decline in the corrosion rate where both the cathodic and anodic partitions of the Tafel plots change to slight values of current density at entirely investigated concentrations. It is obvious that the combination of ZnO/TiO_2_ nanoparticles with the polymer matrix produces noticeable effects on the corrosion current density (*j*_cor_) compared with those confirmed by a pristine polymer. The equivalent cathodic Tafel lines in Figure 6B reveal that the hydrogen evolution was activation-controlled and the cathodic hydrogen development reaction mechanism was not inclined by the presence of the ZnTiO@PANi composite [36]. The corrosion-effective sites on the metal interface are blocked after the adsorption holds happen between the composite additive and the CS-alloy surface. Accordingly, the dipped surface is limited by the hydrogen ions deteriorations, whereas the reaction mechanism relaxations are unchanged.

The values of polarization parameters, including Tafel constant slopes (cathodic *β*_c_ and anodic *β*_a_), *j*_cor_**,** and corrosion potential (*E*_cor_), were calculated, and the results are tabulated in Table 2 as a function of the composite dose. The consistent protection capacity (*η*_t_/%) and the part of the covered surface (*θ*) were computed by the following Equation. [37]:

(6) $$\eta_{p}/\%=\left(\frac{j_{cor,b}-j_{cor,c}}{j_{cor,b}}\right)\times 100=\theta \times 100$$

where *j*_cor,b_ and *j*_cor,c_ indicate the uninhibited and inhibited *j*_cor_, respectively. The examination of Table 2 displays that the *j*_cor_ in the existence of the 100 ppm composite inhibitor is properly smaller (175.03 μA/cm^2^ for individual PAN, 11.15 μA/cm^2^ for ZnTiO@PANi as compared to the blank medium (978.42 μA/cm^2^). When the ZnTiO@PANi inhibitor was introduced into the aggressive medium, the *η*_p_/% improved, and the maximum values of *η*_p_/% were 82.11, and 98.86% at 100 mg L^−1^ for PAN, and ZnTiO@PANi, respectively, indicating that a larger part of the shielded surface is achieved in the aggressive solution with the maximum concentration of ZnTiO@PANi [38], which is protected by the increasing interface coverage (Table 2). It could be obviously recognized from Table 2 that the alterations of cathodic *β*_c_ and anodic *β*_a_ are not unblemished when compared to the blank solution data; this describes that the ZnTiO@PANi composite is adsorbed on the metal surface to reduce the number of active centers on the substrate surface by changing the mechanism of the anode and cathode to hinder the deterioration [39]. The shift magnitude in *E*_cor_ in the occurrence of ZnTiO@PANi composite (>85.0 mV) proposes that it performs as a mixed inhibitor type and affects both cathodic and anodic reactions [40]. The noble shift of *E*_cor_ in the occurrence of the ZnTiO@PANi composite film designates the construction of a combined passive layer on the CS-alloy surface, and as a result, the rate of corrosion (*CR*) decreases. The *CR* of the CS-alloy surface in the presence of 100 ppm of the ZnTiO@PANi composite film was ~ 83.0 orders of magnitude lower than that of CS-alloy in uninhibited solution (77.34 mpy), reaching 0.93 mpy. For the comparison between PANi and ZnTiO@PANi the *η*_p_/% orders are ZnTiO@PANi > PANi.

#### 3.2.2. EIS Studies 

**Figure 7:**
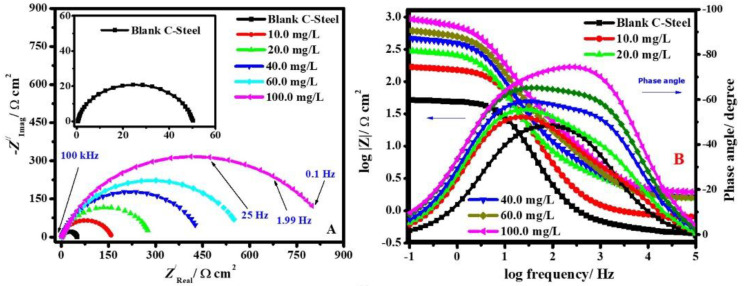
Nyquist (**A**) and Bode modules, (**B**) graphs of CS-alloy corrosion in the molar HCl with 3.5% NaCl and after the addition of diverse concentrations of ZnTiO@PANi at 298 K.

To further understand the impact of the individual PANi and ZnTiO@PANi nanocomposites on corrosion mitigation, EIS measurements were carried out on systems that were both inhibited and uninhibited. The produced polymeric nanocomposites were used for mitigation studies of the CS alloy in an acidic chloride solution by EIS at *E*_OCP_. Nyquist (A) and Bode (B) plots of CS-alloy corrosion in the blank molar HCl containing 3.5% NaCl and adding different doses of ZnTiO@PANi at 298 K are presented in Figure 7A,B, respectively. 

As recognized in Figure 7, Nyquist graphs are shown as a lone semicircle, illustrating that the capacitance of the double layer (*C*_dl_) and charge transfer at the metal/solution interface is what causes the low carbon steel alloy to deteriorate in the studied corrosive solution in the absence and presence of the ZnTiO@PANi compound [41]. The Nyquist plot exhibits a similar style at fully experienced concentrations, illustrating that the addition of a nanocomposite additive to the corrosive media does not affect the corrosion mode [42]. Additionally, as the concentration of additive nanocomposite increases in the experiment medium, the semicircle diameters get wider. The dispersal effect in frequency is evidence that the midpoints of the Nyquist depressed arches were under the real axis [43]. This behaviour may be caused by the metal’s rough surface, the surface’s potential for inhomogeneity, particularly at grain boundaries, and the ZnTiO@PANi nanocomposite additive’s desorption–adsorption process at the steel/electrolyte interaction [44].

The slope of the linear part of the log|*Z*| vs. log(*f*) plots was used to calculate the *α* value, which slows the unevenness of the steel interface (Bode diagram). Theoretically, for a perfect capacitor, the *α* parameter would be (−1). It was noted that the investigated corrosive medium (blank and inhibited systems) *α* parameter for CS-alloy was less than unity [45]. This effect was attributed to metal corrosion in the corrosive solution, which caused the steel substrate to be rough and, as a result, the alloy interface to become inhomogeneous. Additionally, the increase in the phase angle in the occurrence of the ZnTiO@PANI nanocomposite suggests that the capacitive performance of the steel/electrolyte surface is represented by the additive nanocomposite adsorption [46]. The protection proficiency occurred because more ZnTiO@PANI nanocomposites adsorbed at the steel/medium interface, evolving a metal oxide@PANI-Fe complex that was able to shield the CS-alloy interface from corrosion and, as a result, giving rise to a low corrosion rate. This is shown by the Bode phase in Figure 7B, where increasing the dose of ZnTiO@PANI in corrosive solution results in more negative values. The Nyquist modules and Bode phase, respectively, each show one semicircle and one peak. These results point to the existence of a single time constant in the electrochemical pathway because of the binary electrical layer produced at the alloy/medium interface.

**Figure 8:**
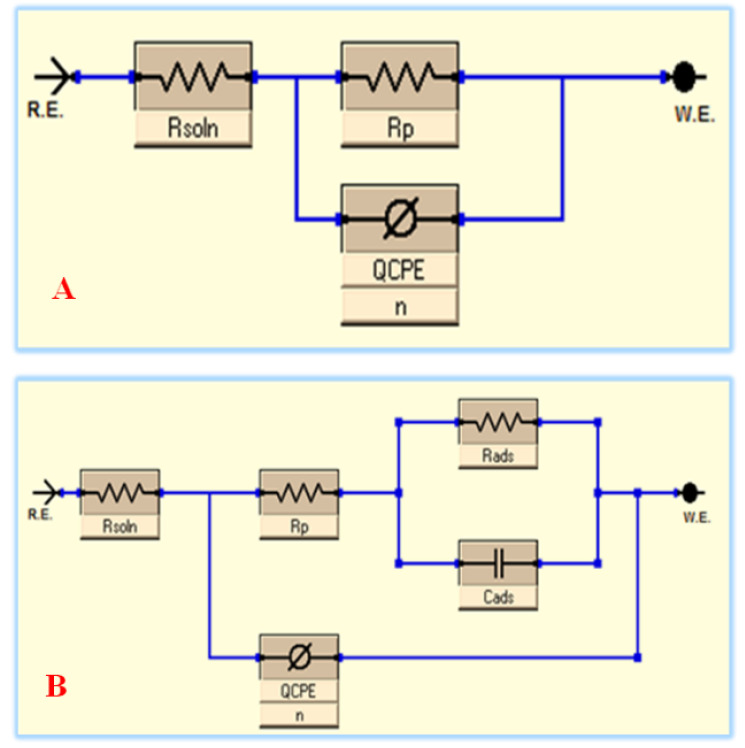
The EEC of unprotected (**A**) and protected systems (**B**).

The comparable circuit instance shown in Figure 8 was utilized to calculate the EIS parameters. The blank system (free inhibitor) results for the systems under study were controlled by the EEC depicted in Figure 8A, while the presence of the 100 ppm ZnTiO@PANI nanocomposite is illustrated in Figure 8B. Table 3 provides a record of the EEC’s appropriateness precision (*χ*^2^). The rather low value of the *χ*^2^ values in Table 3 indicates that our fitting procedure is valid [47].

The element of constant phase (CPE), the solution resistance (*R*_s_), the polarization resistance (*R*_p_), which involves [*R*_p_ = *R*_f_ (film resistance) + *R*_ct_ (charge transfer resistance)], and the parallel of the capacitance of the additive adsorption film (*C*_ads_) and the inhibitor adsorption resistance (*R*_ads_), in the case of the protected system, are all included in this EEC exemplary that fits the EIS strictures. In this case, CPE was used rather than pure capacitance to compensate for the dispersion consequence caused by the surface heterogeneity and roughness of the metal substrate. The following equation provides a clear definition of the CPE impedance (*Z*_CPE_) [48]:

(7) $$Z_{CPE}=\frac{1}{{\left(j\omega \right)}^{n}Y_{0}}$$

where *ω* characterizes the angular frequency, *Y*_o_ signifies the CPE modulus, *j* exemplifies the imaginary, and *n* symbolizes a phase shift. CPE describes an inductor if *n* = −1, a pure resistor if *n* = 0, and a pure capacitor if *n* = 1. According to the findings, we might conclude that adding the ZnTiO@PANi nanocomposite to the aggressive solution causes the water molecules to be substituted and adsorbed on the alloy interface. Interestingly, we noticed that the protected systems’ *n* value, which indicates the degree of heterogeneity, was marginally higher than it was for the uninhibited systems, indicating that the alloys’ interface was relatively more homogeneous. This is due to the homogenous ZnTiO@PANi nanocomposite’s adsorption onto the surface of the metal [49]. The protection capacity (*η*_E_/%) and *C*_dl_ values were calculated as follows [49]:

(8) $$C_{dl}={\left(R_{ct}{}^{1-n}Y_{0}\right)}^{\frac{1}{n}}$$

(9) $$\eta_{E}/\%=\left(\frac{R_{p}^{i}-R_{p}^{0}}{R_{p}^{i}}\right)\times 100$$

where $R_{p}^{i}$ and $R_{p}^{i}$ are the polarization resistance for CS-alloy in blank and containing inhibitors systems, respectively. Table 3 displays that the *R*_p_ values are augmented by an increase in the additive concentration and are higher than those of the unprotected system. The *R*_p_ value augmented from 51.8 to 432.7, and 963.7 Ω cm^2^ when the dose of PANi and ZnTiO@PANi reached 100 ppm, respectively. Consequently, the inhibition capability of 88.0 and 94.6% was achieved, suggesting favourable corrosion protection by ZnTiO@PANi for CS-alloy in the studied corrosive medium. The improvement in *R*_p_ and *η*_E_/% values with an increase in nanocomposite dose is produced by the nanocomposite additives covering a larger portion of the surface, resulting in an alloy corrosion decrease [50]. However, the *C*_dl_ values decreased with an increase in nanocomposite dose compared to the uninhibited system, which shows that additive molecules adsorb on alloy surfaces and cause pre-adsorbed water molecules to dissociate, decreasing the electrode substrate’s electrical capability. Based on Helmholtz’s example, this *C*_dl_ inclination is related to either an increase in the protective layer’s thickness (*d*) or a decrease in the relative dielectric constant (*ε*_c_), as shown in the following Equation. [51]:

(10) $$C_{dl}=\frac{\varepsilon_{c}\varepsilon_{0}}{d}$$

Perfectly, the ZnTiO@PANi adsorption on the metal interface led to an increase in the *d*, and a decrease in the *ε*. As a result, *C*_dl_ for CS-alloy in the inhibitor-containing system appeared to decline. These results show once more that the protection method of ZnTiO@PANi occurs by adsorption onto the electrode surface and are in good agreement with those of the PDP examinations.

### 3.3. DFT Calculations 

**Figure 9:**
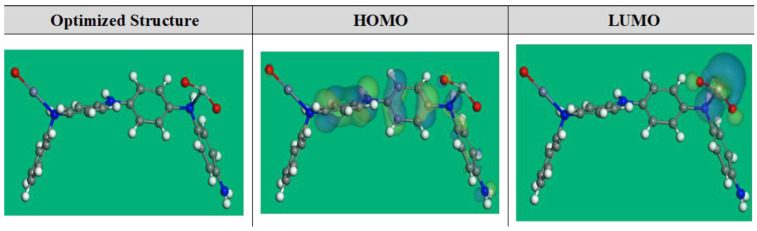
The DFT optimized structure and the occupation of the LUMO and HOMO orbitales for ZnTiO@PANi compound.

To understand the possible LUMO and HOMO energies of the prepared oxide/PANI composite, the DFT optimized structural parameters and correlated theoretical parameters [52] for the ZnTiO@PANi compound were depicted in Figure 9 and listed in Table 4. As per the FMO theory, LUMO and HOMO energies serve to identify the competence of donor or acceptor interactions carried out at the surface of nanocomposite/metal [27]. Therefore, for an inhibitor molecule with low *E*_LUMO_ and high *E*_HOMO_ values, the corrosion inhibition proficiency is augmented. As compared to PANi and ZO@PANi compounds (−4.930 and −4.510 eV), the ZnTiO@PANi molecule has a maximum *E*_HOMO_ value of −4.15 eV, as shown in Table 4. As presented in Figure 9, the level of HOMO was clearly assigned to the phenyl-amino moieties in the additive molecules, indicating that these sites are more likely to be targeted by electrophilic assaults on the CS-alloy surface. These parameters support the capacity of the inhibitor additive to adsorb on the metal interface, leading to an increase in protection effectiveness that was well in line with the empirical results. On the other hand, the *E*_LUMO_ value is −3.00 eV for the ZnTiO@PANi composite (Table 4) lesser than PANi and ZO@PANi compounds (−1.87, −2.03 eV). The greater protective power of the ZnTiO@PANi molecule is indicated by its lower *E*_LUMO_ value, which is consistent with earlier studies.

Correspondingly, the energy gap (Δ*E*) is a significant stricture to improve the corrosion protection effect of the additive molecule, which upsurges as the Δ*E* value is decreased [26,28]. According to Table 4, the ZnTiO@PANi molecule has a stronger propensity to be adsorbed on the steel contact due to its slightly lower energy gap value (1.16 eV) than the PANi and ZO@PANi molecules (3.06 and 2.46 eV). 

Typically, most inhibitors have comparatively low values for electronegativity (*χ*), which represents the inhibitor’s ability to contribute electrons to the surface of the CS-alloy [53]. Contrarily, high values of *χ* also present a great possibility for inhibitor molecules to acquire electrons from iron surface atoms (i.e., back-donation) and form a more durable bond with the CS-alloy surface [54]. As shown in Table 4, it seems that the electronegativity for PANi, ZO@PANi, and ZnTiO@PANi compounds is higher, suggesting that the compounds under consideration have the potential to donate electrons back to one another and form a more stable bond with a CS-alloy interface. 

Additionally, the hardness (*η*) and softness (*σ*) of an inhibitor could be utilized to gauge its reactivity and stability. Soft inhibitors, for example, have a greater shield capacity than hard compounds due to the smooth transfer of electrons to the CS-alloy interface during adsorption, which makes them efficient corrosion inhibitors [55]. Table 4 shows that ZnTiO@PANi molecules have inferior *η* and greater *σ* values than PANi and ZO@PANi molecules, which describes smoothly devoting electrons to the CS-alloy substrate and excellent inhibitory properties.

Likewise, the fraction of electron transfer and Δ*E*_back-donation_ are essential factors for the inhibitor’s proficiency in electron offering or admitting. Consequently, if the Δ*N* values are more than zero, the electron relocation from the inhibitor to the steel interface atoms is likely to occur, but the electron relocation from the metal atoms to the inhibitor molecule is feasible if the Δ*N* values are less than zero (i.e., back-donation) [56]. The fact that the studied molecules had Δ*N* values greater than zero, as listed in Table 4, indicates that the PANi, ZO@PANi, and ZnTiO@PANi molecules are sufficiently able to contribute electrons to the CS-alloy surface. Moreover, the Δ*E*_back-donation_ will be less than zero when *η* is more than zero, the electron will be removed to a compound, followed by a back-donation from the molecule, and this is energetically desired [57]. Table 4 shows that the values of *E*_back-donation_ for PANi, ZO@PANi, and ZnTiO@PANi are negative, indicating that back-donation is desirable for these materials and will result in a strong connection [58].

Furthermore, the dipole moment is a precarious stricture that favors in a predictive method of corrosion protection [59]. The increase in dipole moment improves the adsorption of the compound on the metal substrate and improves the distortion energy. Accordingly, the rise in the dipole moment results in improved anticorrosion ability [60]. As revealed in Table 4, the ZnTiO@PANi compound has a larger value of dipole moment (23.71 Debye) than PANi and ZO@PANi compounds (7.06, 12.72 Debye), which approves the tendency for the ZnTiO@PANi compound to be adsorbed on the metal surface and enhance the protection.

The local reactivity of the prepared compounds can be evaluated by computing the Fukui indices ($f_{k}^{+}$ and $f_{k}^{-}$), Mulliken atomic charges, the local electrophilicity ($\omega_{k}^{\pm }$), local softness descriptor ($\sigma_{k}^{\pm }$), and the dual descriptors ($\Delta f_{k},\Delta \sigma_{k}\;\mathrm{and}\;\Delta \omega_{k}$) from the following equations [61]:

(11) $$\sigma_{k}^{\pm }=\sigma f_{k}^{\pm }$$

(12) $$\omega_{k}^{\pm }=\omega f_{k}^{\pm }$$

(13) $$\Delta f_{k}^{\pm }=f_{k}^{+}-f_{k}^{-}$$

(14) $$\Delta \sigma_{k}=\sigma_{k}^{+}-\sigma_{k}^{-}$$

(15) $$\Delta \omega_{k}=\omega_{k}^{+}-\omega_{k}^{-}$$

**Figure 10:**
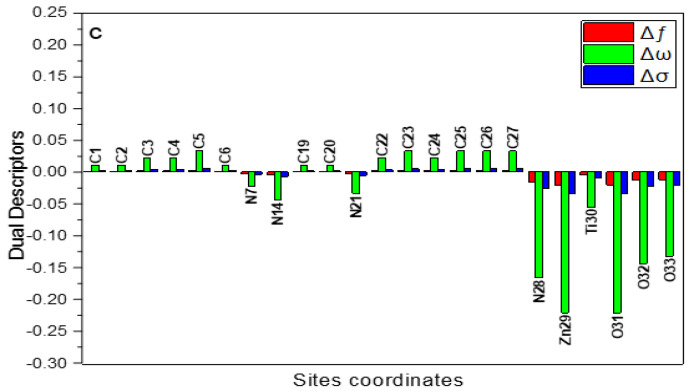
Graphical demonstration of the dual descriptors for the most efficient locations of the examined ZnTiO@PANi compound.

For explanation, Table S1 presents the most important findings. The assessed Fukui indices (Table S1) identified the inhibitor molecules, as well as the places where the molecules of PANi, ZO@PANi, and ZnTiO@PANi will adsorb to the iron surface. The $f_{k}^{+}$ implies the reactivity of the nucleophilic attack centers (accepting centers) whilst $f_{k}^{-}$ designates the electrophilic attack reactivity (donation sites) [62]. The highest $f_{k}^{-}$ for C8-13, C15-20, N14,C22-27, N28 for PANi, C8-13, C15-20, C22-27, N14, N28, O31, Zn30 for ZO@PANi, and C1-5, C8, C10-13, C15-20, C22-26, N14, N28, Ti30, Zn29, and O31-33 for ZnTiO@PANi designating the electron contributing site. While the highest $f_{k}^{+}$ is found at C1-6, C8-13, C15-20, C23-26 for PANi, at C1-6, C8-13, C15-20, C23-24, C26-27, N28, Zn29-30 for ZO@PANi, and at C1-5, C8-13, N14, C15-20, C22-27, N28, Zn29, O31-33, and Si30 for ZnTiO@PANi, revealing the capability for a back-donation [63]. An additional measure of a molecule’s local reactivity is its Mulliken atomic charge, which is shown in Table S1 for the molecules PANi, ZO@PANi, and ZnTiO@PANi. Higher-negative-charged atoms, resemble electron donors (nucleophilic center) [58]. Consequently, the atoms C1-5, N7, C9-10, C12-13, N14, C16-17, C19-20, N21, C23-24, C26-27, N28 for PANi, C1-5, N7, C9-10, C12-13, N14, C16-17, C19-20, N21, C23-24, C26-27, Zn29, N28, O31, O32 for ZO@PANi, and C1-5, N7, C9-10, C12-13, N14, C16-17, C19-20, N21, C23-24, C26-27, O31-33, and N28 for ZnTiO@PANi are active places on oxygen atoms and phenylamino moieties that can provide electrons when they interact with the surface of a metal. Additionally, the local dual descriptors are further accurate tools than the Fukui indices, as well as the local softness and electrophilicity. Figure 10 displays a graphical depiction of the dual local descriptors of the most representative active locations. The attained outcomes displaying that the locations with the $\Delta f_{k},\;\Delta \sigma_{k},\;\mathrm{and}\;\Delta \omega_{k}$< 0 have the propensity to move electrons to the steel interface. In contrast, those sites with $\Delta f_{k},\Delta \sigma_{k}\;\mathrm{and}\;\Delta \omega_{k}$ > 0 have to capability to receive an electron from the CS-alloy. As can be seen in Figure 10, the active centers for electron contribution are at N7, N14, C8, C22-27, N28 for PANi, C22, C24-27, N7, N14, N21, N28, O31, O32, Zn29-30, for ZO@PANi and Zn29, Ti30, N7, N14, N21, N28, and O31-33 for ZnTiO@PANi. While the active centers for electron receiving are at C1-6, C9-13, C15-20 for PANi, C1-6, C8-13, C15-20, C23, C27 for ZO@PANi and C1-6, C19-20, and C22-27 for ZnTiO@PANi.

**Figure 11:**
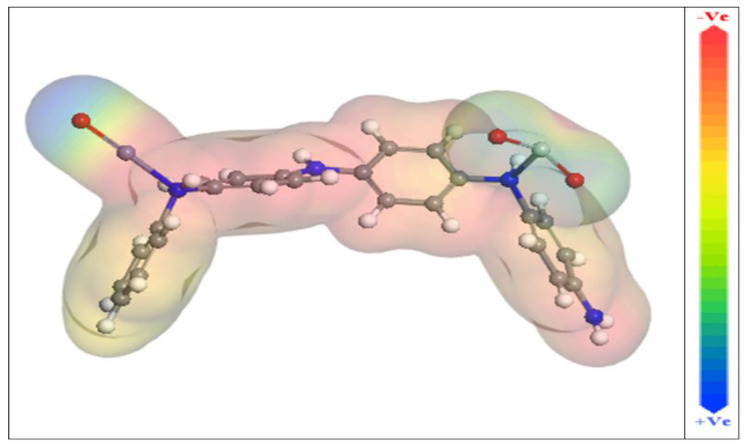
Molecular electrostatic potential (MEP) of the studied and ZnTiO@PANi compound.

Additionally, the Dmol^3^ module evaluates molecular electrostatic potential mapping (MEP), which may reveal the active sites of additive molecules. The MEP mapping is a 3D visual indicator used to identify the total charge distribution’s overall electrostatic influence on a molecule [64]. The great electron density region is where the MEP is particularly negative, is depicted by the red colours in the MEP maps shown in Figure 11 (nucleophilic reaction). Inversely, the shades of blue represent the area with the greatest positivity (electrophilic interaction) [65]. Figure 11’s optical analysis confirms that the highest negative regions are primarily above oxygen and nitrogen atoms, while the benzene rings have a lower electron density. These areas of increased electron density (i.e., the red area) in inhibitor molecules may be the utmost suitable for CS-alloy surface attractions that produce strong adsorbed protective layers.

### 3.4. MC Simulations

**Figure 12:**
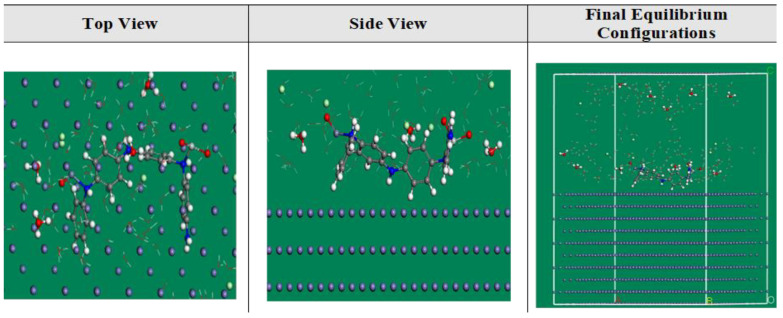
The highest suitable adsorption configuration based on the adsorption locator module for the ZnTiO@PANi compounds on iron (1 1 0) substrate.

In addition to providing a clear notion of the adsorption mechanism, MC simulations were used to identify the attractions of the inhibitor compounds with the metal interface via the adsorption locator module. Figure 12 represents that the inhibitor molecules exist in an approximately flat disposition and achieve the greatest proper adsorption measures for the prepared compounds on the CS-alloy interface in acidic solution. This suggests an improvement in the adsorption and extreme covered surface [66]. Additionally, Table 5 reveals the calculated results based on MC simulations for the adsorption energies. It was discovered that the ZnTiO@PANi molecule (−2113.72 kcal mol^−1^) has a higher negative value of adsorption energy than the PANi (−1831.61 kcal mol^−1^) and ZO@PANi −1938.63 kcal mol^−1^) which assumes energetic adsorption of ZnTiO@PANi on the metal substrate, constructing a stable adsorbed layer, and shielding the CS-alloy interface from corrosion. These results are consistent with the results of the experiment [67]. Additionally, Table 5 exhibits that the value of adsorption energy for the ZnTiO@PANi compound for the pre-geometry optimization stage, i.e., unrelaxed (−1712.61 kcal mol^−1^), is more-negative than individual polymer and ZO@PANi compounds (−1533.32, −1589.97 kcal mol^−1^) and for the post-geometry optimization stage i.e., relaxed (401.33) are greater than PANi and ZO@PANi compounds (−298.41, −273.63 kcal mol^−1^), confirming a higher inhibition capability for ZnTiO@PANi composite than other compounds. 

If adsorbed H_2_O or an inhibitor molecule is not included, the d*E*_ads_/d*N*_i_ values help to clarify the steel/adsorbates conformation energy [26]. According to Table 5, the ZnTiO@PANi molecule exhibits superior adsorption over PANi and ZO@PANi molecules because its d*E*_ads_/d*N*_i_ value (−321.06 kcal mol^−1^) is higher than those of PANi and ZO@PANi molecules (−219.81, −273.21 kcal mol^−1^). Additionally, for water molecules, hydronium ions, and chloride ions, the d*E*_ads_/d*N*_i_ values are approximately −17.44, −54.53, and −95.83 kcal mol^−1^, respectively. These values are small when compared with the values of PANi molecules, ZO@PANi and ZnTiO@PANi composites, which reveal that inhibitor molecules adsorb strongly relative to H_2_O molecules, chloride ions, and hydronium ions, thereby promoting the inhibitor compounds’ superiority over these ions. According to both experimental and theoretical investigations, the PANi, ZO@PANi, and ZnTiO@PANi compounds are therefore stubbornly adsorbed on the CS-alloy interface and create a potent adsorbed defensive film that leads to corrosion protection for CS-alloy surface in the corrosive medium.

### 3.5. Comparative Studies with Previous Reports

This research of nanocomposite based on PANI functionalized ZnO_2_-TiO_2_ NPs is the first of its type, where it is used as a protective film for CS-alloy in a corrosive solution of acidic containing chloride. Comparative tests of the inhibition capabilities of ZnTiO@PANi composites with other polymeric materials utilized as inhibitors for acidic steel corrosion are reported in Table 6 [4,68,69,70,71,72]. It is observed that the efficiency of the ZnTiO@PANi nanocomposite is the greatest for CS alloy in the studied corrosive media at 100 ppm compared to other materials.

## 4. Conclusions

In this work, we synthesized a novel nanocomposite based on a polyaniline functionalized ZnO-TiO_2_ nanoparticle in order to study as a corrosion protection film; the conclusions drawn are the following:

1. The SEM and TEM descriptions confirmed that the fabricated ZnTiO@PANi composite has a heterogeneous morphology containing crystalline (oxide) and amorphous (polymer) parts. Moreover, the XPS analysis approves that the surface chemistry of the synthesized nanocomposite has C as a major content in addition to O, N, Zn, and Ti.
2. The produced nanocomposite (ZnTiO@PANi) exhibits sufficient acidic inhibitory efficacy. The maximal dose of 100 mg/L of PANi and ZnTiO@PANi gave protective capabilities of 82.11 and 98.86%, respectively.
3. The PDP method revealed that the addition of the ZnTiO@PANi composite material leads to a noticeable decrease in the *j*_cor_ and both cathodic and anodic slopes. This result shows the mixed type of ZnTiO@PANi composite.
4. According to the EIS results, the polarization resistance values (*R*_p_) increased with a higher dose of the ZnTiO@PANi compound than in the blank system, confirming a greater degree of protection provided by the addition of the nanocomposites.
5. The protective ability, as assessed by empirical testing, was related to the theoretical findings. Using MC and DFT studies, further information was discovered on the reactivity centers and adsorption energies for the attraction of ZnTiO@PANi on the Fe (110) interface, respectively.
6. In short, this study presented a novel ZnTiO@PANi composite as a powerful acidic corrosion inhibitor with the study of many characterization tools and DFT studies to understand the chemistry and mechanism of inhibition.

## Figures and Tables

**Table 1:** The chemical composition of the used CS-alloy in corrosion experiments.

Element	C	Cr	Mn	P	S	Si	Fe
**Percentage/%**	0.29	0.15	0.88	0.034	0.039	0.18	98.607

**Table 2:** Potentiodynamic restrictions of CS-alloy corrosion in the blank molar HCl containing 3.5% NaCl and with the addition of diverse doses ZnTiO@PANi at 298 K.

Systems	*C*_inh._/ mg/L	*J*_cor_/ µA cm^−2^	*CR/*mmp ±SD	*−E*_cor_/ mV (Ag/AgCl)	*β*_a_/ mV dec ^−1^	−*β*_c_/ mV dec −1	*θ*	*η*_t_/%
Blank	0.0	978.42	77.34 ± 5.3	444 ± 25	72.83	167.38	-	-
PANi	100	175.03	13.84 ± 1.1	456 ± 29	78.39	170.65	0.821	82.11
ZnTiO@PANi	10	568.35	44.93 ± 3.7	436 ± 21	71.32	170.93	0.419	41.91
	20	356.52	28.23 ± 2.4	439 ± 15	75.66	166.44	0.635	63.56
	40	183.37	14.54 ± 1.5	438 ± 12	79.52	168.04	0.812	81.26
	60	58.50	4.64 ± 0.5	440 ± 19	77.54	167.18	0.940	94.02
	100	11.15	0.93 ± 0.06	438 ± 20	78.77	171.24	0.988	98.86

**Table 3:** EIS restrictions for the corrosion of CS-alloy in the acidic chloride solution and after the addition of diverse doses ZnTiO@PANi at 298 K.

Additive Codes	*C*_inh._/ mol/L	*R*_s_/ Ωcm^2^	*R*_P_/ Ωcm^2^ ±SD	*C*_dl_/ Fcm^−2^ × 10^−6^	*Q*_CPE_		*χ^2^* × 10^−4^	*θ*	*η*_E_/%
					*Y*_0_/ μΩ^−1^ s^n^ cm^−2^	*n*			
**Uninhibited**	0.0	0.54	51.8 ± 3.6	460.81	58.58	0.743	5.41	--	--
**PANi**	100	1.2	432.7 ± 34.1	82.43	9.01	0.837	4.54	0.880	88.0
**ZnTiO@PANi**	10	1.15	166.1 ± 14.1	147.07	28.11	0.838	5.32	0.688	68.8
	20	2.71	293.3 ± 21.6	121.46	20.17	0.848	4.93	0.823	82.3
	40	3.23	463.4 ± 35.8	76.09	14.82	0.874	5.82	0.888	88.8
	60	3.79	615.6 ± 42.4	40.97	10.83	0.868	5.45	0.915	91.5
	100	4.07	963.7 ± 39.7	24.22	8.73	0.865	4.53	0.946	94.6

**Table 4:** DFT parameters of the studied compounds.

Parameters	PANi	ZO@PANi	ZnTiO@PANi
*E*_HOMO/_eV	−4.93	−4.51	−4.15
*E*_LUMO/_eV	−1.87	−2.03	−3.00
Δ*E*= *E*_LUMO_ − *E*_HOMO_/eV	3.06	2.46	1.16
µ/Debye	7.06	12.72	23.71
*η*	1.53	1.25	0.58
∆*E*_back-donation_	−0.38	−0.31	−0.14
ω	3.76	4.28	11.06
Δ*N*	1.19	1.51	2.96
*χ*	3.41	3.28	3.57
*σ*	0.66	0.81	1.73

**Table 5:** Information and parameters derived from MC simulations of the PANi, ZO@PANi, and ZnTiO@PANi compounds adherent to iron (1 1 0).

Corrosion Systems	Adsorption Energy/ kcal mol^−1^	Rigid Adsorption Energy/ kcal mol^−1^	Deformation Energy/ kcal mol^−1^	d*E*_ads_/dN_i_: Inhibitor kcal mol^−1^	d*E*_ads_/d*N*_i_: Cl^−^ Ions kcal mol^−1^	d*E*_ads_/d*N*_i_: HYDRONIUM kcal mol^−1^	d*E*_ads_/d*N*_i_: Water kcal mol^−1^
Fe (110)	−1831.61	−1533.32	−298.41	−219.81	−95.62	−54.05	−17.26
PANi							
Water							
Hydronium							
Cl^-^ ions							
Fe (110)	−1938.63	−1589.97	−348.63	−273.21	−96.12	−54.90	−17.76
ZO@PANi							
Water							
Hydronium							
Cl^-^ ions							
Fe (110)	−2113.72	−1712.61	−401.33	−321.06	−95.76	−54.65	−17.29
ZnTiO@PANi							
Water							
Hydronium							
Cl^-^ ions							

**Table 6:** Comparative studies with previous reports.

Compound	Alloys\Metal	Inhibitor Concentration	Methods Used	Protection Capacity/%	References
ZnTiO@PANi	CS-alloy	100 mg/L^−1^	*E*_ocp_, EIS, and PDP	98.8	Present work
Tl_2_O_3_-SiO_2_/PANI	C-steel	10-150 mg/L^−1^	EIS, PDP, *E*_OCP_, HRTEM, and SEM	52.4–93.8	[4]
polyaniline–nano-TiO_2_ composites	steel	13–41.8% of TiO_2_ in product	*E*_OCP_, SEM, TEM	92.1	[59]
Epoxy/polyaniline–ZnO nanorods	steel	1.3–4.9% of ZnO in product	*E*_OCP_, SEM, and EIS	94.3	[60]
PANI/Ag	Al1050 alloy	Coated film	CV, SEM, and EIS	97.5	[61]
PANI/Zn	Al1050 alloy	Coated film	CV, SEM, and EIS	92.5	[61]
Fe_2_O_3_/PPy	Mild steel	Coated film	EIS and *E*_OCP_	91.6	[62]
Epoxy/MgO NPs coatings	Mild steel	Coated film	SEM	93.7	[63]
polyaniline functionalized ZnO-SiO_2_ nanoparticles	CS-alloy	150 ppm	EIS and PDP	98.6	[22]

## Data Availability

The raw/processed data generated in this work are available upon request from the corresponding author.

## Supplementary Materials

The following supporting information can be downloaded at: https://www.mdpi.com/article/10.3390/ma15217589/s1. Figure S1: A representative scheme of the fabrication route of ZnO nanoparticles; Figure S2: A representative scheme of the fabrication route of ZnO-TiO_2_ nanoparticles; Figure S3: A representative scheme of the fabrication route of binary MO/PANi nanocomposites by oxidation in situ-polymerization; Table S1: The evaluated Fukui indices.
