# Supplementary material for: Synthesis and Characterization of the Mixed Metal Oxide of ZnO-TiO2 Decorated by Polyaniline as a Protective Film for Acidic Steel Corrosion: Experimental, and Computational Inspections
Source: Materials (Basel). 2022 Oct 28;15(21):7589. doi: 10.3390/ma15217589 (PMC9653851; doi:10.3390/ma15217589)
Supplement: Supplementary file 1 [file materials-15-07589-s001.zip › materials-1932073-supplementary.pdf]

materials

MDPI

Supplementary Materials

Synthesis and characterization of the mixed metal oxide of ZnO-TiO_2 decorated by polyaniline as a protective layer for acidic steel corrosion: Experimental, and Computational inspections

May Ahmed Al-Masoud [1], Mai M. Khalaf [1,2], Mohamed Gouda [1], Van-Duong Dao [3,*], Ibrahim M. A. Mohamed [2], Kamal Shalabi [4,5] and Hany M. Abd El-Lateef [1,2,*]

1 Department of Chemistry, College of Science, King Faisal University, Al-Ahsa 31982, Saudi Arabia
2 Department of Chemistry, Faculty of Science, Sohag University, Sohag 82524, Egypt
3 Faculty of Biotechnology, Chemistry and Environmental Engineering, Phenikaa University, Hanoi 10000, Vietnam
4 Department of Chemistry, College of Science and Humanities in Al-Kharj, Prince Sattam bin Abdul-Aziz University, Al-Kharj 11942, Saudi Arabia
5 Chemistry Department, Faculty of Science, Mansoura University, Mansoura 35516, Egypt
* Correspondence: hmahmed@kfu.edu.sa or hany_shubra@science.sohag.edu.eg (H.M.A.E.-L.); duong.daovan@phenikaa-uni.edu.vn (V.-D.D.)

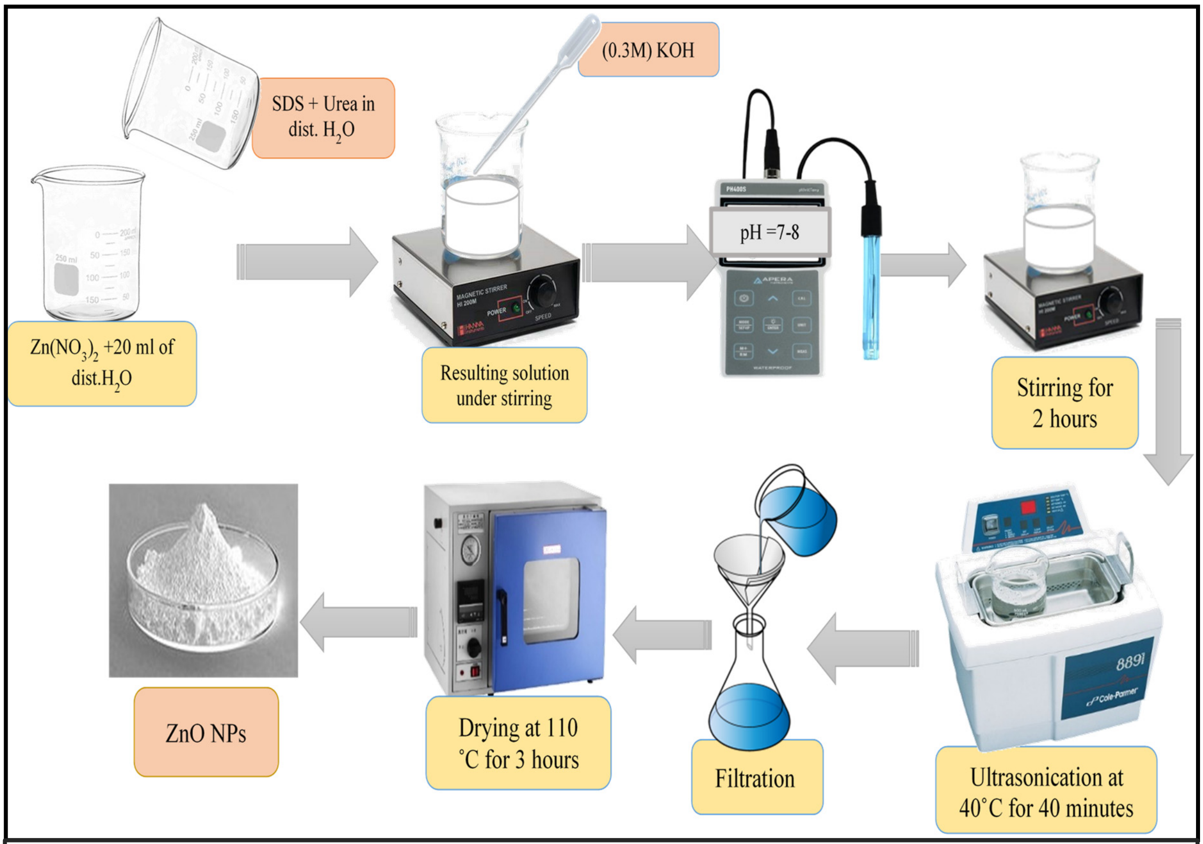

Figure S1. A representative scheme of the fabrication route of ZnO nanoparticles.

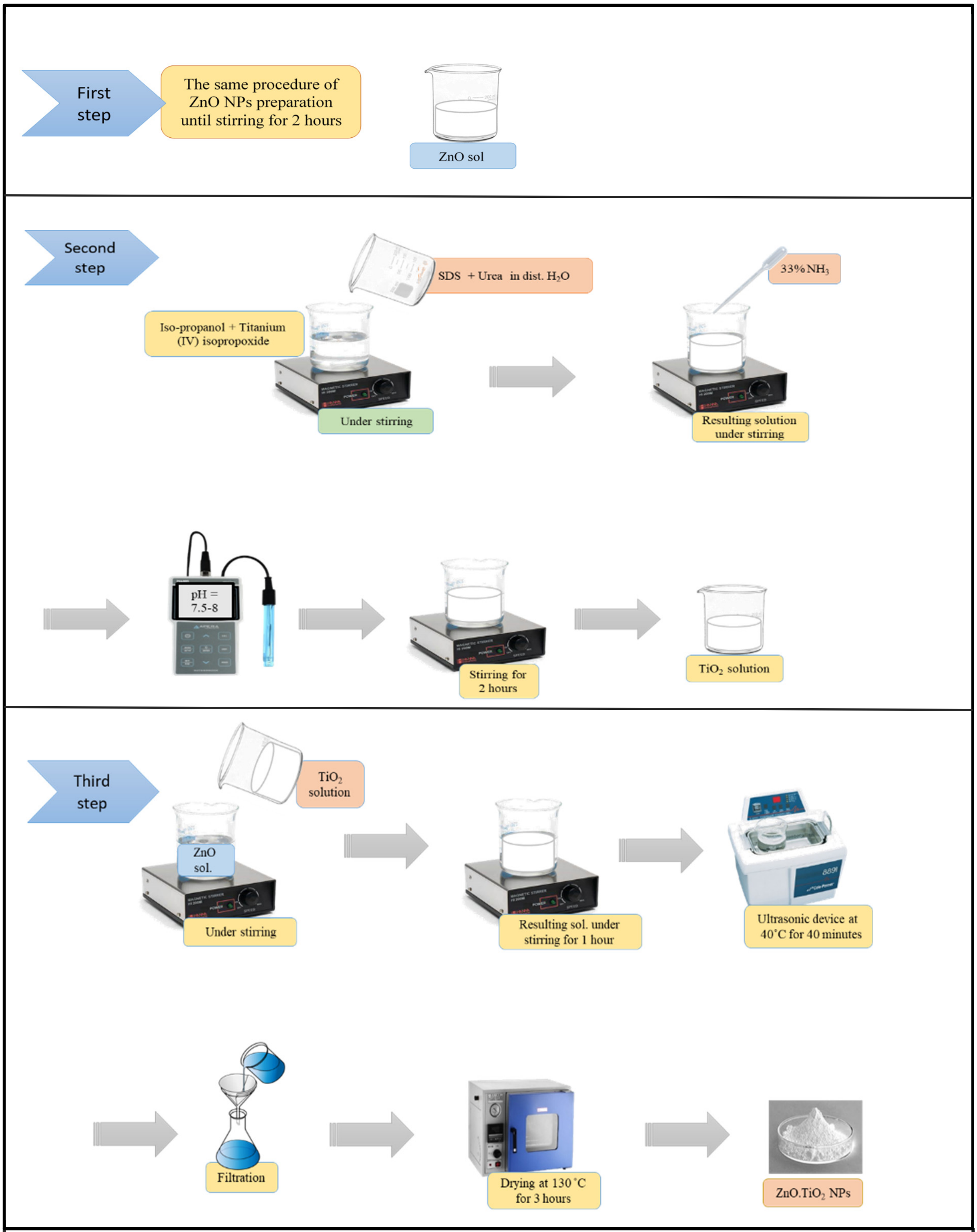

Figure S2. A representative scheme of the fabrication route of ZnO-TiO_2 nanoparticles.

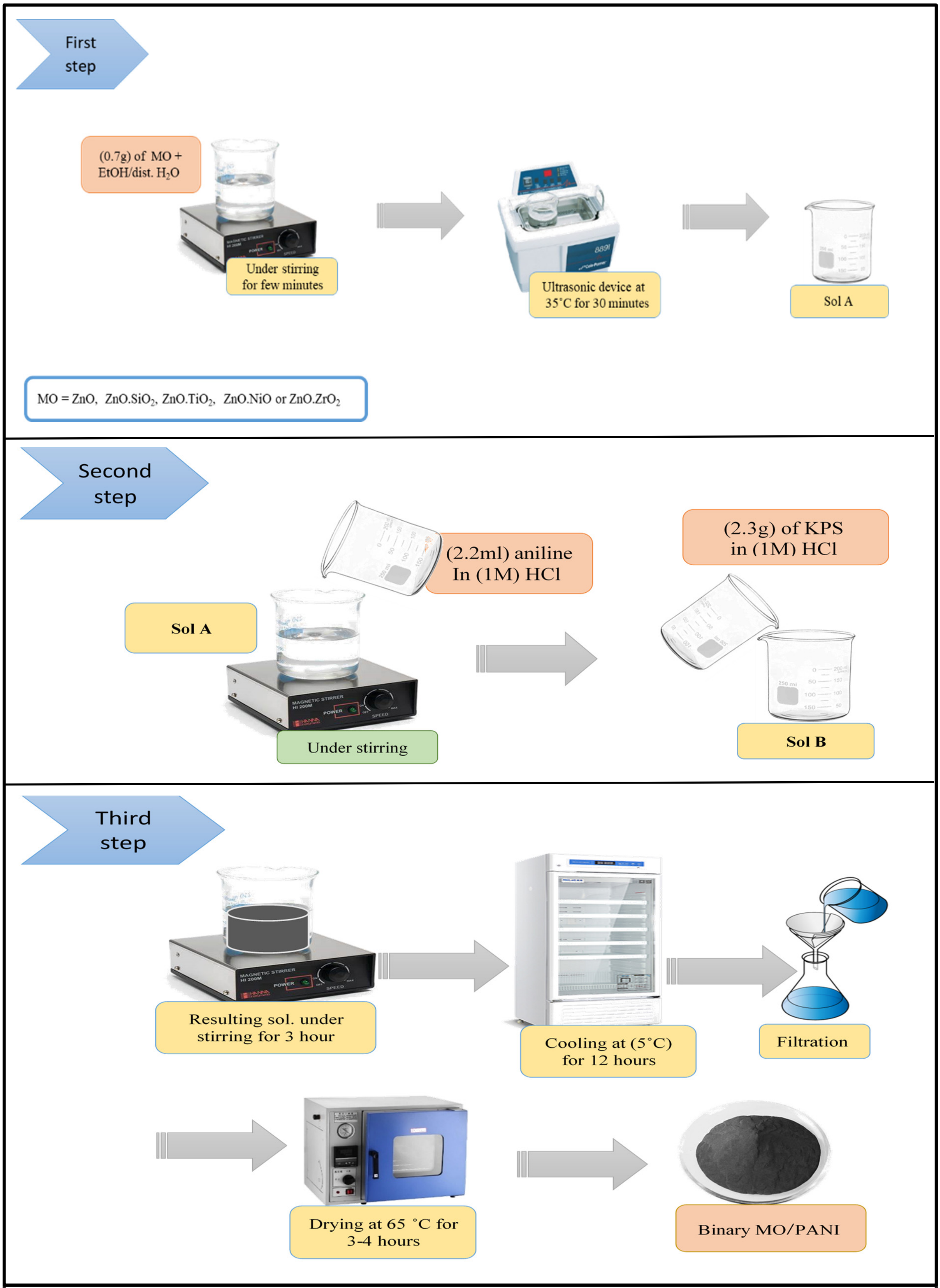

Figure S3. A representative scheme of the fabrication route of binary MO/PANi nanocomposites by oxidation in situ-polymerization.

Table S1. The evaluated Fukui indices.

Atom no	PANi						
	f+	ω+	σ+	f-	ω-	σ-	Mulliken Charges
C1	0.027	0.101	0.018	0.005	0.019	0.003	-0.087
C2	0.026	0.098	0.017	0.005	0.019	0.003	-0.043
C3	0.030	0.113	0.020	0.006	0.023	0.004	-0.045
C4	0.024	0.090	0.016	0.005	0.019	0.003	-0.042
C5	0.030	0.113	0.020	0.006	0.023	0.004	-0.091
C6	0.019	0.071	0.012	0.001	0.004	0.001	0.203
N7	-0.004	-0.015	-0.003	-0.001	-0.004	-0.001	-0.244
C8	0.023	0.086	0.015	0.025	0.094	0.016	0.169
C9	0.032	0.120	0.021	0.014	0.053	0.009	-0.083
C10	0.034	0.128	0.022	0.026	0.098	0.017	-0.101
C11	0.027	0.101	0.018	0.011	0.041	0.007	0.222
C12	0.028	0.105	0.018	0.022	0.083	0.014	-0.130
C13	0.033	0.124	0.022	0.015	0.056	0.010	-0.086
N14	0.008	0.030	0.005	0.046	0.173	0.030	-0.270
C15	0.023	0.086	0.015	0.014	0.053	0.009	0.223
C16	0.027	0.101	0.018	0.026	0.098	0.017	-0.102
C17	0.030	0.113	0.020	0.016	0.060	0.010	-0.082
C18	0.022	0.083	0.014	0.019	0.071	0.012	0.169
C19	0.025	0.094	0.016	0.016	0.060	0.010	-0.079
C20	0.028	0.105	0.018	0.026	0.098	0.017	-0.135
N21	-0.002	-0.008	-0.001	-0.002	-0.008	-0.001	-0.242
C22	0.005	0.019	0.003	0.041	0.154	0.027	0.148
C23	0.012	0.045	0.008	0.019	0.071	0.012	-0.079
C24	0.018	0.068	0.012	0.048	0.180	0.031	-0.124
C25	0.007	0.026	0.005	0.018	0.068	0.012	0.231
C26	0.017	0.064	0.011	0.044	0.165	0.029	-0.122
C27	0.013	0.049	0.008	0.021	0.079	0.014	-0.081
N28	0.02	0.075	0.013	0.098	0.368	0.064	-0.352

Atom no	ZnO@PANi						
	f+	ω+	σ+	f-	ω-	σ-	Mulliken Charges
C1	0.024	0.103	0.019	0.005	0.021	0.004	-0.096
C2	0.023	0.098	0.018	0.005	0.021	0.004	-0.067
C3	0.027	0.116	0.022	0.006	0.026	0.005	-0.069
C4	0.020	0.086	0.016	0.004	0.017	0.003	-0.066
C5	0.028	0.120	0.022	0.006	0.026	0.005	-0.092
C6	0.017	0.073	0.014	0.002	0.009	0.002	0.162
N7	-0.011	-0.047	-0.009	-0.009	-0.039	-0.007	-0.301
C8	0.017	0.073	0.014	0.015	0.064	0.012	0.114
C9	0.031	0.133	0.025	0.013	0.056	0.010	-0.082
C10	0.028	0.120	0.022	0.020	0.086	0.016	-0.114
C11	0.025	0.107	0.020	0.011	0.047	0.009	0.184
C12	0.030	0.128	0.024	0.022	0.094	0.018	-0.159
C13	0.027	0.116	0.022	0.011	0.047	0.009	-0.086
N14	0.007	0.030	0.006	0.031	0.133	0.025	-0.237
C15	0.024	0.103	0.019	0.011	0.047	0.009	0.177
C16	0.029	0.124	0.023	0.019	0.081	0.015	-0.112
C17	0.030	0.128	0.024	0.013	0.056	0.010	-0.080
C18	0.023	0.098	0.018	0.017	0.073	0.014	0.128
C19	0.027	0.116	0.022	0.011	0.047	0.009	-0.094
C20	0.028	0.120	0.022	0.020	0.086	0.016	-0.155
N21	-0.005	-0.021	-0.004	-0.004	-0.017	-0.003	-0.298
C22	0.003	0.013	0.002	0.017	0.073	0.014	0.114
C23	0.014	0.060	0.011	0.010	0.043	0.008	-0.093
C24	0.014	0.060	0.011	0.021	0.090	0.017	-0.120
C25	0.008	0.034	0.006	0.011	0.047	0.009	0.148
C26	0.015	0.064	0.012	0.021	0.090	0.017	-0.121
C27	0.013	0.056	0.010	0.011	0.047	0.009	-0.084
N28	0.013	0.056	0.010	0.044	0.188	0.035	-0.368
Zn29	0.044	0.188	0.035	0.199	0.851	0.160	-0.015
Zn30	0.037	0.158	0.030	0.136	0.582	0.109	0.015
O31	0.001	0.004	0.001	0.010	0.043	0.008	-0.550
O32	0.001	0.004	0.001	0.006	0.026	0.005	-0.555

Atom no	ZnTiO@PANi						
	f+	ω+	σ+	f-	ω-	σ-	Mulliken Charges
C1	0.009	0.100	0.016	0.008	0.088	0.014	-0.091
C2	0.009	0.100	0.016	0.008	0.088	0.014	-0.062
C3	0.013	0.144	0.022	0.011	0.122	0.019	-0.076
C4	0.011	0.122	0.019	0.009	0.100	0.016	-0.065
C5	0.013	0.144	0.022	0.010	0.111	0.017	-0.087
C6	0.004	0.044	0.007	0.003	0.033	0.005	0.156
N7	-0.011	-0.122	-0.019	-0.009	-0.100	-0.016	-0.586
C8	0.018	0.199	0.031	0.018	0.199	0.031	0.172
C9	0.007	0.077	0.012	0.007	0.077	0.012	-0.100
C10	0.021	0.232	0.036	0.021	0.232	0.036	-0.075
C11	0.010	0.111	0.017	0.010	0.111	0.017	0.164
C12	0.018	0.199	0.031	0.018	0.199	0.031	-0.121
C13	0.012	0.133	0.021	0.012	0.133	0.021	-0.055
N14	0.030	0.332	0.052	0.034	0.376	0.059	-0.167
C15	0.014	0.155	0.024	0.014	0.155	0.024	0.158
C16	0.022	0.243	0.038	0.022	0.243	0.038	-0.078
C17	0.013	0.144	0.022	0.013	0.144	0.022	-0.073
C18	0.013	0.144	0.022	0.013	0.144	0.022	0.160
C19	0.010	0.111	0.017	0.009	0.100	0.016	-0.058
C20	0.018	0.199	0.031	0.017	0.188	0.029	-0.120
N21	-0.013	-0.144	-0.022	-0.010	-0.111	-0.017	-0.615
C22	0.025	0.277	0.043	0.023	0.254	0.040	0.156
C23	0.014	0.155	0.024	0.011	0.122	0.019	-0.059
C24	0.027	0.299	0.047	0.025	0.277	0.043	-0.09
C25	0.014	0.155	0.024	0.011	0.122	0.019	0.136
C26	0.028	0.310	0.048	0.025	0.277	0.043	-0.084
C27	0.009	0.100	0.016	0.006	0.066	0.010	-0.078
N28	0.05	0.553	0.087	0.065	0.719	0.112	-0.219
Zn29	0.07	0.774	0.121	0.09	0.995	0.156	0.875
Ti30	0.05	0.553	0.087	0.055	0.608	0.095	1.523
O31	0.06	0.664	0.104	0.08	0.885	0.138	-0.756
O32	0.03	0.332	0.052	0.043	0.476	0.074	-0.723
O33	0.031	0.343	0.054	0.043	0.476	0.074	-0.721
